# The Impact of Control Measures and Holiday Seasons on Incidence and Mortality Rate of COVID-19 in Iran

**DOI:** 10.34172/jrhs.2020.35

**Published:** 2020-12-06

**Authors:** Saiedeh Haji-Maghsoudi, Majid Sadeghifar, Ghodratollah Roshanaei, Hossein Mahjub

**Affiliations:** ^1^Department of Biostatistics, School of Public Health, Hamadan University of Medical Sciences, Hamadan, Iran; ^2^Department of Statistics, Faculty of Basic Sciences, Bu-Ali Sina University, Hamadan, Iran; ^3^Modeling of Noncommunicable Diseases Research Center, Hamadan University of Medical Sciences, Hamadan, Iran; ^4^Research Center for Health Sciences, Hamadan University of Medical Sciences, Hamadan, Iran.

**Keywords:** Autoregressive Hidden Markov Model (ARHMM), Holiday seasons, Control measures, COVID-19

## Abstract

**Background:** Preventive measures on the COVID-19 pandemic is an effective way to control its spread. We aimed to investigate the effect of control measures and holiday seasons on the incidence and mortality rate of COVID-19 in Iran.

**Study design:** An observational study.

**Methods:** The daily data of confirmed new cases and deaths in Iran were taken from the Johns Hopkins University COVID-19 database. We calculated weekly data from 19 Feb to 6 Oct 2020. To estimate the impact of control measures and holiday seasons on the incidence rate of new cases and deaths, an autoregressive hidden Markov model (ARHMM) with two hidden states fitted the data. The hidden states of the fitted model can distinguish the peak period from the non-peak period.

**Results:** The control measures with a delay of one-week and two-week had a decreasing effect on the new cases in the peak and non-peak periods, respectively (*P*=0.005). The holiday season with a two-week delay increased the total number of new cases in the peak periods (*P*=0.031). The peak period for the occurrence of COVID-19 was estimated at 3 weeks. In the peak period of mortality, the control measures with a three-week delay decreased the COVID-19 mortality (*P*=0.010). The expected duration of staying in the peak period of mortality was around 6 weeks.

**Conclusions:** When an increasing trend was seen in the country, the control measures could decline the incidence and mortality related to COVID-19. Implementation of official restrictions on holiday seasons could prevent an upward trend of incidence for COVID-19 during the peak period.

## Introduction

The new coronavirus outbreak was initiated from the Wuhan, Hubei Province, People's Republic of China, on Dec 29, 2019. The coronavirus (SARS-CoV-2) as a member of a family of relatively large viruses is responsible for COVID-19 ^1^. The infectious disease spreads through a population via the specific transmission routes of COVID-19^2,3^. Since the emergence of COVID-19 until 28 Nov 2020, 62048552 people have been infected and 1450146 related deaths have been reported in the world ^4^.

The pandemic of COVID-19 has had a tremendous impact on the health; financial, physical, and mental status in the world ^5^. No vaccine or specific effective anti-viral drug regime is yet available for this disease ^6-8^. For this reason, many policymakers and decision-makers have focused their prevention and control policies on non-pharmacological methods ^9^. Quarantine, social distancing, school and university closures, and travel restrictions are some of the measures to reduce mixing in society ^10-13^. It is important for governments to monitor the progress of disease and control this epidemic.

In Iran, the first cases of COVID-19 were reported in Feb 2020 ^4^. Following the outbreak of this disease in the country, extensive measures were taken to control this disease. These included schools and universities closure ^14^, developing various awareness-raising programs to encourage people to stay home, controlling travel, reducing working hours, setting teleworking schedules ^15^, sports clubs and swimming pools closure ^16^. Moreover, Friday prayers and the other religious gatherings were closed ^16^. With the onset of the New Year in Iran in the third decade of Mar, as intercity travel increased, concerns grew for health policy-makers. Therefore a policy of social distancing, as well as maximum restrictions on traveling, was widely implemented in Iran from the late Mar, 2020^15^.

Despite the increasing number of infected people for COVID-19 and its mortality rate in the world, there is no sufficient scientific evidence about the disease and accurate effect of control measures. Currently, controlling this disease is one of the most important health concerns in the world ^17^. The effect of travel restrictions and control measures on the spread of COVID-19 have been studied by different research^18^ - ^21^. In Iran, the effect of social distance on COVID-19 has been evaluated generally ^22^.

A proper understanding of the dynamics of this disease greatly enhances its control and prevention. However, the unique features of the prevalence of COVID-19 have limited applications of existing models ^23^.

There are different methods to model the data, one is Hidden Markov models (HMMs). What distinguishes HMMs apart from other methods is the modeling of data with dynamic behavior that takes into account the effect of unobserved variables. Ordinary time series models such as autoregressive moving average (ARMA) or interrupted time series models do not have such a feature. These models have been used in various fields so far. Some applications include disease mapping ^24^, diagnosis of influenza epidemics ^25^, hospital infection data ^26^, forecasting COVID-19 cases ^27^, and investigation of the psychosocial impact of COVID-19 lockdown on mental wellbeing ^28^. The HMMs can incorporate the dynamics characteristics of the COVID-19 transmission in the model. The dynamic feature is related to mechanisms that cause infection change over time. These mechanisms are not directly visible. Some reasons for changing the mechanism of the disease are safety and quarantine measures, mutations in the virus, immunity of individuals over time, and seasonal factors ^10,29-32^.

 In the present study, an analytical method called HMOs was used. Based on the literature, a comprehensive study was not found to evaluate the government restrictions and holiday seasons' impact on the intensity spread of COVID-19 and death using HMM. So, we were able to evaluate the effect of the studied variables under different epidemic and non-epidemic conditions. Therefore, we aimed to investigate the impact of some factors on the occurrence of COVID-19 in Iran using HMMs.

## Methods

In this observational study, the daily data of COVID-19 related to the total confirmed new cases and deaths in Iran from 19 Feb to 6 Oct 2020 were taken from an online dataset ^33^. We collected holiday seasons from the calendar. Holiday seasons are days when people are more inclined to travel due to a few days off. Furthermore, according to the instructions of the Iranian Corona Headquarters, severe restrictions were imposed by the government for about a month from the late Mar 2020 throughout the country ^15^, we named it government restrictions or control measures. Most of these measures are related to the imposition of restrictions such as the closure of shopping malls, stores, parks, and recreation centers, restrictions on long-distance travel, and minimal presence of office staff. The factors of holiday seasons and control measures used for modeling of weekly COVID-19 data in Iran.

The weekly data of confirmed new cases and new deaths, based on positive PCR results, were analyzed separately using the autoregressive hidden Markov model (ARHMM). The holiday seasons and control measures were the observed covariates in the applied model. HMMs are structurally composed of two models: the measurement model and the hidden model. In this type of modeling, the observations are influenced by a hidden chain ^34^. This model enables us to take into account the nature of correlated data. The combination of the hidden Markov chain and autoregressive time series creates the ARHMM. By applying ARHMM, the observations sequence is separated by hidden states ^35^.

In this model, smoothing probabilities represent the probability of being in each state at time t based on available observations. Furthermore, the impact of each covariate was obtained under each state. The *P*-value less than 0.05 was considered as statistical significance. Data analysis and programming were performed using R 4.0.2 and Stata 14.2 (Stata Corp, College Station, TX, USA) software.

## Results

 In Iran during the study period, the total number of confirmed cases and deaths were 479825 and 27405, respectively. The minimum and maximum daily confirmed cases per million people were 0.02 and 46.32, respectively. The daily reported deaths per million people due to COVID-19 were 0.01 and 2.79. The mean (SD) was 24.44 (10.05) per million for daily cases and 1.40 (0.70) per million for deaths. Moreover, the median (interquartile range) for new cases and deaths was 26.57 (12.75) and 1.44 (1.07), respectively. Furthermore, the coefficient of variation for daily confirmed cases and deaths was 41.13% and 50.39%, respectively.

**Figure 1:**
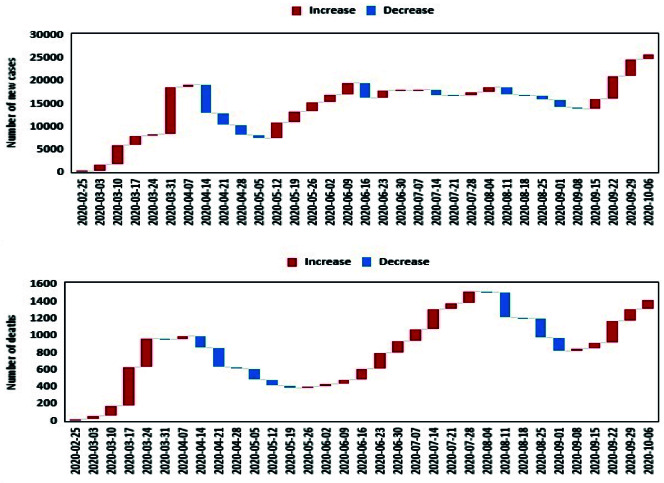
The trend of weekly changes related to reported new cases and deaths of COVID-19 in Iran from 19 February to 6 October 2020. Top panel: Trend of weekly changes related to the number of reported new cases of COVID-19. Bottom panel: Trend of weekly changes related to the number of reported deaths of COVID-19.

The top panel of Figure 1 shows the weekly changes in the reported number of new cases from 19 Feb to 6 Oct 2020 in Iran. The bottom panel of Figure 1 presents the reported number of deaths due to COVID-19 during the time. At the beginning of the epidemic, in less than two months, the number of new cases reached the highest level in early Apr. The most changes were seen from 25 Mar to early Apr. During this period, an increasing trend with a steep slope was observed. Another upward trend is from late May to early June. In the days leading up to 6 Oct, another upward trend appeared. The most declining changes are seen in two consecutive weeks in mid-April. The first peak period of death was observed in mid-Mar to mid-Apr, after which we again see the second peak of deaths from the disease in Aug. A new upward trend in mortality appears to have begun in Sep.

**Figure 2:**
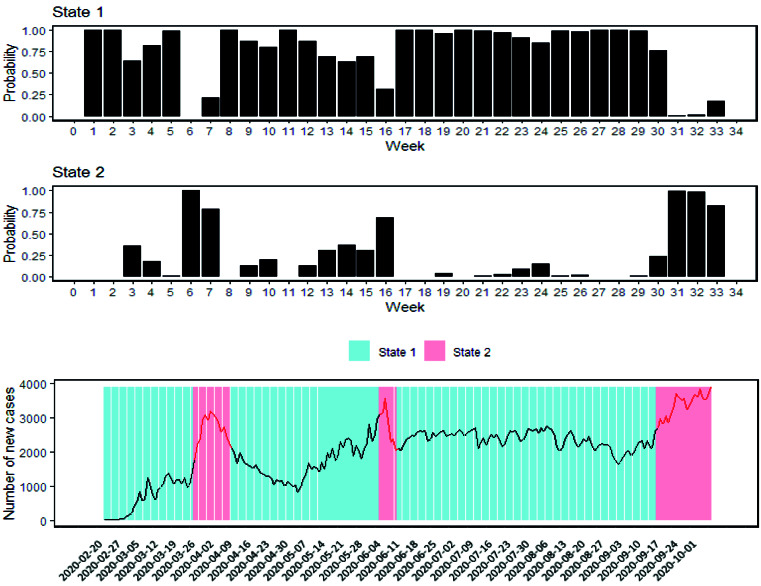
State specification of new cases of COVID-19 in Iran using ARHMM (Two top panels: Smoothed probabilities of being each state during the time. Bottom panel: Estimated states of the hidden process.).

The hidden state specification of the new cases data was presented in Figure 2. Two top panels of this figure show the smoothing probabilities for each state. The bottom panel presents the states of the hidden process during the time. Based on the figure, state 2 is representative of the peak period.

The estimation results of ARHMM for weekly new cases for each state were reported in Table 1. Initially, at the top of the table, the coefficients related to the effect of each variable on the new cases with standard error (S.E.) and *P*-value were reported separately for each state. Below that, the estimation of transition probabilities was displayed. The last part of this table displays the expected duration of each state.

Control measures with a delay of two-week decrease the incidence of COVID-19 in the non-peak period (*P*=0.005). Besides, in the peak period, control measures have more impact on the decrease of the new cases with a delay of one-week (*P*=0.005). The holiday seasons increase the new cases with a delay of two-week in the peak period (*P*=0.031). The impact of control measures with a delay of three-week is not significant in none states (Table 1).

**Table 1:** Estimated parameters of ARHMM for the incidence rate of COVID-19

**Variables**	**Coefficient**	**SE**	***P*** **-value**
State 1 (Non-peak period)			
Intercept	27.15	11.91	0.023
New cases with a delay of one-week	0.88	0.05	0.001
Holiday seasons with a delay of two-week	-10.69	9.80	0.275
Control measures with a delay of one-week	-3.03	19.32	0.875
Control measures with a delay of two-week	-58.29	20.69	0.005
Control measures with a delay of three-week	25.79	15.87	0.104
State 2 (Peak period)			
Intercept	54.41	48.97	0.267
New cases with a delay of one-week	0.86	0.19	0.001
Holiday seasons with a delay of two-week	56.39	26.13	0.031
Control measures with a delay of one-week	-74.05	26.47	0.005
Control measures with a delay of two-week	11.23	54.00	0.835
Control measures with a delay of three-week	-1.58	48.03	0.974
**Expected duration**	**Estimate**	**SE**	
State 1	7.83	8.09	-
State 2	2.57	1.75	-
**Transition probabilities**	**State 1**	**State 2**	
State 1	0.87	0.13	-
State 2	0.39	0.61	-

The probability of staying in the non-peak period is 0.87. This state is fairly persistent. The value of 0.13 indicates the probability of being in the peak period in the next week given that the hidden process is in the non-peak period in the current week. Furthermore, 0.61 displays the probability of staying in the peak period given that the process is in the same state in the current week. The transition probability from peak to non-peak period is 0.39. The expected duration of staying in the non-peak and peak period is 7.83 and 2.57 weeks, respectively (Table 1).

**Figure 3:**
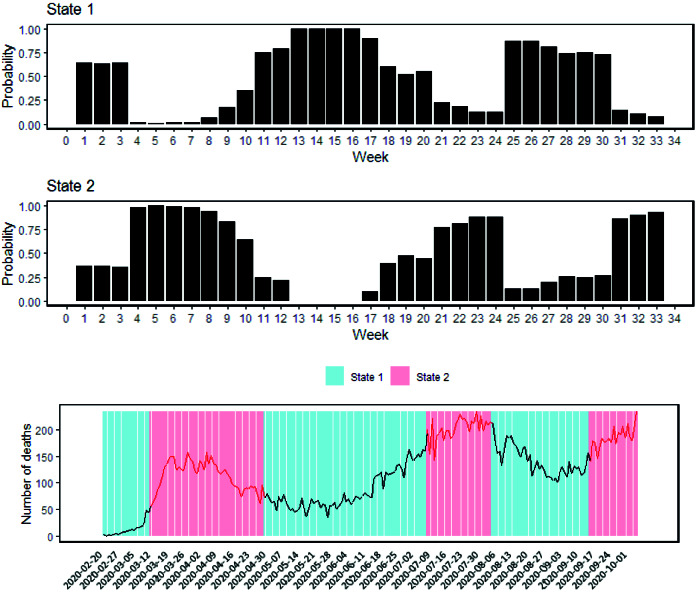
State specification of new deaths due to COVID-19 in Iran using ARHMM (Two top panels: Smoothed probabilities of each state during the time. Bottom panel: Estimated states of the hidden process.)

The smoothing probabilities and hidden state periods for new deaths were presented in Figure 3. State 2 is representative of a peak period in deaths.

The results of ARHMM for weekly new deaths show that there is an association between new deaths and control measures in peak periods. The control measures with a delay of three-week decrease the new deaths during the peak period (*P*=0.001) (Table 2).

 The probability of staying in the non-peak period is 0.86 given that staying in the same state in the current week. The probability of staying in the peak period is 0.83 given that staying in the same state in the current week.

The transition probability from peak to non-peak period is 0.17. Moreover, the transition probability from non-peak to peak period is 0.14. The expected duration of staying in the non-peak and peak period is 7.03 and 6.06 weeks, respectively (Table 2).

**Table 2:** Estimated parameters of ARHMM for mortality rate due to COVID-19

**Variables**	**Coefficient**	**SE**	***P*** **-value**
New deaths with a delay of one-week	0.89	0.06	0.001
State 1 (Non-peak period)			
Intercept	12.1	3.15	0.001
Holiday seasons with a delay of two-week	-0.40	0.73	0.584
Control measures with a delay of three-week	-0.03	1.28	0.980
Control measures with a delay of four-week	-0.57	1.11	0.611
Control measures with a delay of five-week	-0.77	0.99	0.434
State 2 (Peak period)			
Intercept	15.92	3.09	0.001
Holiday seasons with a delay of two-week	-1.30	0.77	0.092
Control measures with a delay of three-week	-3.91	1.51	0.010
Control measures with a delay of four-week	-0.13	1.51	0.930
Control measures with a delay of five-week	-0.78	2.32	0.737
**Expected duration**	**Estimate**	**SE**	
State 1	7.03	4.43	-
State 2	6.06	3.61	-
Transition probabilities	State 1	State 2	
State 1	0.86	0.14	-
State 2	0.17	0.83	-

## Discussion

 Based on the results, official control measures are an effective way to decrease the trend of infected people for COVID-19 and related deaths. This policy can be more effective when the epidemic and its death are in peak periods. Moreover, holiday seasons increase the trend of the epidemic for peak periods. We separated the peak periods for epidemic and related deaths by ARHMMs. The ARHMM was an effective tool to discover the dynamic behavior of COVID-19 in Iran. The effects of investigated variables were estimated under each hidden state. The expected duration of staying in the non-peak period for new cases was more than triple compared to the peak-period. Government restrictive measures in both states can be helpful to reduce the spread of the virus. When the country is at the peak period of the epidemic, it is possible to control the epidemic by imposing restrictions on unnecessary travel during the holiday seasons. Of course, the impact of these measures can be seen with a delay of two weeks for holiday seasons and a one-week delay for government restrictive measures.

 The expected duration of staying in the non-peak period of mortality due to COVID-19 is near to two months. Whereas, the peak-period is more than a month. Both states are fairly persistent. This means the probability of transition to another state is low. This can be welcomed for the non-peak period of mortality because if the country is in the non-peak period, it will stay in this state for a relatively long time if keep other factors. On the other hand, it is unpleasant for the peak period. When it is in a peak period of mortality, it will stay in peak for a relatively long time. In the peak period of mortality, it is possible to reduce the trend of mortality by establishing restrictive regulations, since the impact of control measures was significant only in the peak period.

In Iran, social distancing policy was an effective strategy to control the spread of COVID-19. This policy had led to a decrease in new cases and deaths in Iran. An upward trend was seen before the implementation of social distancing ^22^. Their findings are consistent with the results of the present study.

One study in the US estimated the economic benefit of social distancing. 1.7 million lives can be saved by the implementation of moderate social distancing. The findings confirmed the substantial economic benefit of social distancing policies ^36^. In India, a three-week lockdown was not enough to prevent resuscitation. Instead, stable lockdown protocols with periodic relaxation are recommended. Due to these measures, they have predicted a reduction in morbidity and mortality ^37^. Moreover, delay in implementation of control measures can lead to high morbidity and mortality ^38,39^. These results in line with our findings.

In China, the control strategies in social mixing were effective tools to reduce the incidence and mortality of COVID-19 in Chinese cities ^13,38^. Another study in the US was conducted to evaluate the effect of social distancing on COVID-19 in different states of the US. Their finding showed it reduces the spread of the new coronavirus ^40^. These findings are consistent with our results.

 The strength of the present study is to quantify the effect of control measures and holiday seasons on the incidence and mortality of COVID-19 underlying peak and non-peak periods. However, the investigated factors in this study were limited to control measures and holiday seasons while the upward and downward of the epidemic and related deaths are affected by many factors. Some of them are improvement in health care facilities, diagnostic tests, providing certain medications, and making masks mandatory in public places. On the other side, at the beginning of the epidemic, there was widespread panic throughout the country, which led to observe health protocols by people, while in recent months, its effect has been less visible. It is suggested to investigate the effect of some other related factors for future works.

## Conclusions

 Holiday seasons with a two-week delay increase the incidence of COVID-19 and restrictive regulations by the government are effective factors to control the epidemic with a one-week delay for the peak period and a two-week delay for the non-peak period. Government intervention measures can also be effective to reduce mortality due to COVID-19. In general, official control measures can play a valuable role in reducing morbidity and mortality of COVID-19. This means the implementation of official government restrictions along with public health interventions could reduce the morbidity and mortality of COVID-19. Furthermore, preventing unnecessary travels during holiday seasons could reduce the spread of this disease.

## Acknowledgments

 We would like to thank the Vice-Chancellor for Research and Technology of Hamadan University of Medical Sciences, Iran. This study is a part of the Ph.D. thesis of the first author in Hamadan University of Medical Sciences, Iran with an approved Ethical code: IR.UMSHA.REC.1396.648.

## Conflict of interest

 The authors declare that they have no conflict of interest.

## Funding

 This work was supported by Hamadan University of Medical Sciences, Iran (No 9609286096).

## Highlights

- The control measures are effective to control the incidence and mortality of COVID-19.
- The impact of control measures in the peak period is more than in the non-peak period.
- In the peak period, the holiday seasons lead to an upward trend of COVID-19.
- The expected duration of the peak period for the incidence of COVID-19 was 2.57 weeks.
- The expected duration of the peak period for mortality was around 6 weeks.
